# Beyond supply and demand: a new ecological framework for understanding the access of young migrants to sexual and reproductive health services in Sweden

**DOI:** 10.1080/16549716.2023.2251783

**Published:** 2023-09-12

**Authors:** Mazen Baroudi

**Affiliations:** Department of Epidemiology and Global Health, Umeå University, Umeå, Sweden

**Keywords:** Culture, quality of healthcare, sexual education, cultural sensitivity, youth

## Abstract

**Background:**

Although the sexual and reproductive health and rights (SRHR) of young people and migrants should be prioritised, young migrants’ sexual and reproductive health (SRH) is rarely studied in Sweden.

**Objectives:**

To explore young migrants’ understanding and experiences of sexual rights and examine their perceptions and experiences in accessing SRH services.

**Methods:**

This is a mixed method study including a national survey that recruited 1773 newly arrived young migrants; a youth clinic survey that recruited 1089 youths after visiting youth clinics; and a qualitative study that included 13 interviews with newly arrived Arabic-speaking migrant men. The results are synthesised using a new ecological framework of access to understand the factors influencing young migrant access to health care based on the levels of the ecological model and the five steps of access: approachability, acceptability, adequacy, affordability, and quality.

**Results:**

Young migrants understood SRH as both ‘essential’ and ‘a right.’ Their sexual rights were less fulfiled compared to other young people in Sweden, particularly for men, non-binary, LGBTQ+, those born in South Asia, without a residence permit, and those of low economic conditions. SRH services were largely unapproachable as almost half of those who needed them did not utilise them. Services were generally acceptable due to the ‘open environment,’ however, some young migrants faced cultural insensitivity, fear of exposure, low parental support, and long waiting times. SRH services’ quality was perceived as good, however, negative experiences were reported, particularly in the domains of respect, equity, privacy, non-prejudice, and consultation quality.

**Conclusion:**

The access of young migrants to SRH services is facilitated by an ‘open environment’ and available and good quality services; however, they faced serious barriers such as limited access to information about the health system, comprehensive sexual education, lack of cultural sensitivity, and cultural racism.

## Introduction

Migrants tend to have worse sexual and reproductive health (SRH) outcomes with higher rates of maternal morbidity and mortality, higher risk of HIV/AIDS, and greater exposure to sexual violence [1–5]. One explanation for these worse outcomes is the lower access to SRH services faced by migrants which can be due to both demand and supply factors.

On the demand side, some norms and stigma around SRH might play a role in affecting health-seeking behaviours and access to SRH services, for example, some migrant women might avoid services without female healthcare providers (HCPs) [6,7]. Other demand factors include socio-economic status, lack of access to information, health literacy, language skills, and mistrust in the health system [8–10].

On the supply side, legal entitlement can also limit access to SRH services, for instance, just a handful of countries within the European Union provide equal access to healthcare for undocumented migrants, and even when entitlements are in place, they are often not known or respected in practice [11,12]. In addition, cultural racism and a lack of cultural sensitivity from HCPs can contribute to the poorer SRH experiences of migrants [7,13].

Worse SRH outcomes for migrants can also be attributed to structural factors outside the healthcare system, for example, structural violence affects migrants’ access to resources such as employment and housing, influencing their overall health including SRH [14].

### Young migrants and sexual and reproductive health and rights (SRHR) in Sweden

SRHR inequities persist in Sweden, based on factors such as age, gender, country of origin, education level, functionality, and sexual orientation [15–17]. Migrants in Sweden face significant SRHR challenges, including higher rates of HIV diagnosis (70% of cases), late-stage diagnosis, and limited access to SRHR services. Furthermore, migrants are more likely to feel a lack of respect in healthcare [18,19]. Similarly, young people in Sweden are also exposed to SRHR challenges, including risky sexual behaviours, exposure to sexual violence, and declining rates of condom usage [16,20]. These challenges have led to increased incidences of sexually transmitted diseases (STDs), unplanned pregnancies, and abortions [20].

According to the Swedish SRHR strategy, both young people (those aged 16–29 years old) and migrants (those who were born outside Sweden) face heightened SRHR challenges and need stronger SRHR [21]. Young migrants face barriers to accessing healthcare and information about their rights, leading to marginalisation and discrimination [2,22,23]. The access of young migrants to healthcare is lower compared to non-migrant peers and older migrants in Sweden [24]. Subgroups of young migrants, such as those belonging to the LGBTQ+ communities, have further increased risks of SRHR violations, which have a negative impact on their sexual agency and overall health and well-being [2,25]. The likelihood of sexual violence and infringements on sexual rights are higher among young migrant women, asylum seekers, and undocumented migrants [2,26,27]. Additionally, there has been a growing awareness of the sexual exploitation and violence encountered by unaccompanied boys and men [28,29].

### Migration and migration policies in Sweden

Statistics Sweden (the official statistics bureau of Sweden) defines a migrant as ‘foreign-born,’ meaning anyone who moves to Sweden, intends to stay for at least 12 months, and is registered in the population register [30]. Migration patterns in Sweden have shifted from labour market migration to asylum seeking and family reunions, resulting in most recent migrants coming from low- and middle-income countries. The proportion of foreign-born in the Swedish population has increased from 15% to 20% in the last two decades, with a rise in young migrants, particularly from non-European countries like Syria, Iraq, Afghanistan, Somalia, Eritrea, Thailand, and Iran [31]. The 2010s saw a rise in nationalist politics and anti-migrant sentiment in the popular discourse [32]. While Sweden had a long history of generous migration policies with a high proportion of migrants receiving permanent residence and a high possibility for family reunification, the migration policies in Sweden changed after the 2015 ‘migration crisis.’ It became more difficult to obtain residence permits and temporary permits became a standard hindering family reunification, especially for unaccompanied minors (68).

### Health policies and healthcare organization in Sweden

Healthcare in Sweden is regulated by the Healthcare Act (2017:30) and the Public Health Policy (2007/08:110) which prioritise healthcare on equal terms. Other acts, including the Patients Act (2014:821), the Discrimination Act (2008:567), and the Infection Control Act (2004:168), aim to promote equal rights and prevent discrimination in healthcare and SRHR services. The basic principle of these acts is equal healthcare for equal needs without discrimination [33].

Sweden’s national strategy for SRHR was published in 2020 with the overall aim of providing good and equal SRH to the whole population. The strategy recommends several measures to reach its objectives including creating structural conditions to improve SRHR, showing SRHR as part of public health, and ensuring equal and accessible care, support, and treatment. The strategy identifies certain populations that need to have their SRHR strengthened, including ‘young people, people with migration backgrounds, people with inadequate socio-economic conditions, people with disabilities, and the LGBTQ+ community’ [21].

The Swedish healthcare system is publicly funded through taxes and social insurance, with the National Board of Health and Welfare of Sweden (Socialstyrelsen) accountable for creating guidelines while county councils deliver the healthcare services. Certain SRH services such as antenatal, intrapartum, postnatal care, contraception, abortion, and treatment of sexually transmitted diseases are available free of charge or heavily subsidised to all people in Sweden, regardless of their residency status. Other SRH services are covered by social insurance for residents only. These services include, for example, reproductive cancer treatment, trans-specific healthcare, and infertility treatment. Asylum seekers receive a free introductory health assessment on arrival in Sweden to inform them about the Swedish health system and their health needs [34,35].

SRH services in Sweden include primary care, such as youth clinics and health centres, and specialised care, such as maternity units, fertility care, and STD clinics. Youth clinics (ungdomsmottagningar) are a network of health services targeting young people up to 22–30 years of age and offer mainly preventive SRH services along with mental healthcare and other services [36].

### The focus and rational of the thesis

Despite the prioritised public responsibility for the right to health, including SRH, for all residents in Sweden, there is little information about how young migrants access SRH services in the country [37,38]. National surveys like UngKAB15 and SRHR17 do not address the context of young migrants and are only sent to registered migrants and in Swedish, leaving out asylum seekers and undocumented migrants, which makes it challenging to arrive at conclusions about young migrants’ SRH from these surveys [15,16].

Although individual papers have targeted different migrant groups as detailed in Table 1, the thesis focuses on young (those 16–29 years old), newly arrived migrants (arrived to Sweden in the last ten years), particularly those from low- and middle-income countries (according to the World Bank’s classification). Migrants’ conditions in the hosting countries contribute to making newly arrived migrants more vulnerable to SRH problems and having more challenges in access to healthcare compared to more established migrants [21,39,40].

**Table 1.** Summary of the four papers comprising this paper. Adapted from [53].

	Design	Target population and sampling approach	Data collection	Data analysis and objectives
National survey	Population-based cross-sectional study.	1773 newly arrived (less than ten years), young (16–29 years old), migrants born in low- and middle-income countries, and resident in all Swedish counties. Recruited through visits to schools and other arenas, distributing letters through SCB, or using a web survey.	Self-administered survey in 2018	In Paper 1, descriptive statistics were used to depict young migrants’ sexual rights.
				In Paper 2, multivariate multiple linear regression was used to evaluate young migrants’ access to SRH services, explore the barriers they encounter, and compare different sociodemographic groups.
Youth clinic survey	Clinic-based cross-sectional study.	1089 youth (16–25 years old) visiting one of 20 youth clinics located in the four northern counties of Sweden. Including first- and second-generation migrants from outside Scandinavia.	Self-administered survey in 2016/17	Employing a multi-level analysis, Paper 3 aimed to investigate the perceptions of youth clinics’ friendliness among migrants and Swedish/Scandinavian youth.
Qualitative study	Interview-based qualitative study.	13 young (22–37 years old) newly arrived (less than ten years) Arabic-speaking migrant men. Recruited through social media announcements and snowball sampling.	Semi-structured interviews in 2020/21	Using qualitative thematic analysis, Paper 4 explored the perceptions and experiences of Arabic-speaking migrant men of information, education, and services related to SRH.

Due to the sequential explanatory mixed methods approach, the results of the quantitative part informed the qualitative part to place special focus on men. Young men take more sexual risks, have more sexual ill health, and face more barriers in accessing SRH services due to gender norms and expectations. This hinders their access to SRH services and organisational barriers that include a generally low priority of men’s SRH and SRH services, which are women centred [41–43].

The thesis uses an SRHR lens and focuses on sexual rights and access to SRH services. The attainment of the sustainable developmental goals is contingent on the recognition of sexual rights as an integral aspect of human rights [44,45]. Access to SRH services for all plays a critical role in achieving universal health coverage and the highest standard of SRH [46,47]. The thesis also highlights the importance of providing youth-friendly health services that are engaging, sensitive, and effective [48,49].

The thesis aimed to study SRH among young migrants in Sweden, exploring their understanding and experiences with sexual rights, as well as examining their perceptions and experiences in accessing SRH services.

## Conceptual framework

The thesis focuses on two concepts: sexual rights and access to SRH services. Sexual rights is conceptualised and operationalised based on the Guttmacher – Lancet Commission report [45]. The definition provided by the report is grouped into five domains, including the right to: 1) access SRH services; 2) access information and sexual education; 3) body integrity free from coercion and violence; 4) make free, informed decisions about sexual relations and sexuality; and 5) a safe and satisfying sexual life without stigma and discrimination. Several indicators reflecting each of the domains were explored.

**Figure 1.**
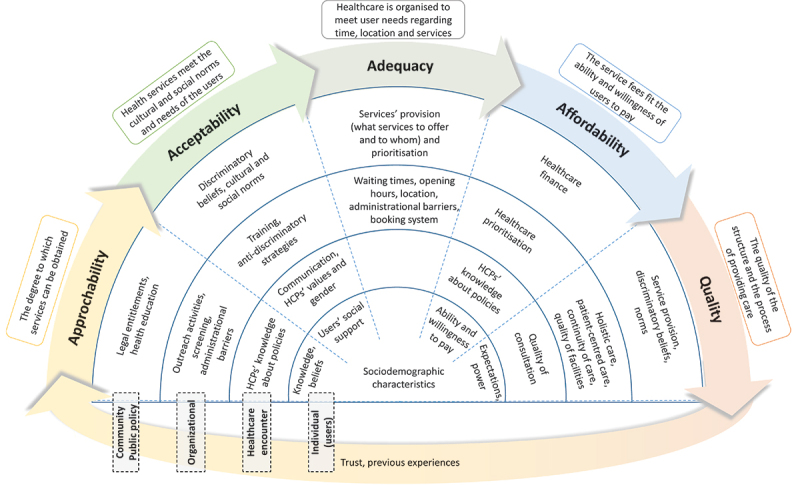
Ecological framework of access to healthcare. Reprinted from [53].

After conducting a literature review on access to conceptualisation and the factors that impede or enhance healthcare access, the thesis proposes an ecological framework for healthcare access. The framework is inspired by previous frameworks of access including the Levesque and Obrist frameworks and the ecological model [50–52]. This framework views access as a process that involves five steps: approachability, acceptability, adequacy, affordability, and quality. Approachability refers to the degree to which healthcare services are known to users and can be obtained by them. Acceptability refers to the extent to which healthcare services meet the social and cultural norms and needs of users. Adequacy is viewed as the organisation of healthcare to meet users’ needs in terms of time, location, and services. Affordability refers to the service fees fitting the ability and willingness of the users to pay. Quality refers to the structure and process of providing care, including the quality of the facility and the consultation (Figure 1).

Drawing inspiration from the socio-ecological model of health, the framework is significant as it redirects attention from the healthcare service users to the healthcare organisation, public health policies, and the community, making explicit the areas where the responsibility for enhancing access to healthcare lies. Furthermore, approaching access as a process allows for a comprehensive interpretation of the factors related to healthcare access and facilitates more direct connections to policy implications. This is in contrast to the common risk-based approach, which only identifies populations at risk without offering significant guidance for policy and practice considerations [54]. The framework is non-linear, as access is influenced by previous encounters and the degree of trust in the healthcare system. These factors consequently impact future access to healthcare. However, the framework does not account for the impact of structural factors such as housing, living, and working conditions, which may affect young migrants’ need to access healthcare services.

The framework with its steps is used to study the various barriers to accessing SRH services among young migrants. The views and perspectives of healthcare providers and policy makers regarding migrant access to healthcare are not addressed in this thesis.

## Methods

### Research process

I used a sequential explanatory mixed methods approach [55], starting with quantitative data followed by qualitative data collection and analysis. The results of the quantitative studies informed the focus of the qualitative study on migrant men and SRH services. The thesis comprises four papers that are based on three studies including a national survey, youth clinic survey, and a qualitative study. Table 1 provides a summary of the individual papers. The first two papers are based on a national survey commissioned by the Swedish Public Health Agency, focusing on young migrants’ sexual rights and access to SRH services [56,57]. Paper 3 is based on a youth clinic survey conducted in northern Sweden, exploring migrants’ perceptions of the friendliness of youth clinics [58]. Paper 4 is a qualitative study that focuses on young migrant men’s perceptions and experiences of SRH services and sexual information/education [59]. This paper integrates the results of the quantitative and qualitative studies to provide a detailed picture of young migrants’ access to SRH services in Sweden.

### The national survey (Papers 1 & 2)

The national survey of young migrants in Sweden targeted individuals aged 16–29 years old who were born outside of Sweden and lived in Sweden without consideration to their residence status. Data were collected in 2018 and involved visits to Swedish language schools, secondary schools with language introduction programmes, and migrants’ associations, distributions of letters through Statistics Sweden (SCB), and promotion of a web survey on social media. The questionnaire used was developed in Swedish and English based on existing national and international questionnaires [15,16,60] and was translated and back-translated between English and Arabic, Tigrinya, Dari, and Somali after a pilot study in a Swedish language school to ensure language and cultural appropriateness. A total of 1773 participants were included in the study. The response rate was 81% in the face-to-face data collection, 15% in the letters sent by SCB, and is not measurable in online data collection. Over two thirds of the respondents were male and over 50% of them were born in the Middle East and North Africa (Table 2). The data were analysed using descriptive statistical analysis and multivariate multiple linear regression using Stata 15 statistical software. The first paper analysed the indicators of sexual rights, stratifying the data by region of birth, residence permit, sexual orientation, and gender. The second paper used the framework of access of Levesque et al. [51] to measure access to SRH services in four stages: SRH service needs, perceptions of needs, utilisation, and met needs. The prevalence of the four stages of access was estimated while controlling for age, region of birth, gender, education level, economic status, residence permit, and reason for migration.

**Table 2.** Sample characteristics of the national survey. Reprinted from [53].

		n (%)
Gender		
	Women	586 (34.9)
	Men	1060 (63.1)
	Non-binary	34 (2.0)
Region of birth		
	Middle East & North Africa	989 (57.4)
	South Asia	313 (18.2)
	Sub-Saharan Africa	370 (21.5)
	Other	51 (3.0)
Sexual orientation		
	Heterosexual	1134 (73.1)
	LGBA	201 (13.0)
	Don’t want to answer	217 (14.0)
Residence permit		
	Waiting	188 (11.6)
	2016 or later	911 (56.0)
	Before 2016	528 (32.5)
Age		
	16–19 years	670 (37.8)
	20–25 years	592 (33.4)
	26–29 years	511 (28.8)
Reason of migration		
	Asylum	1108 (72.2)
	Family reunion	313 (20.4)
	Work	46 (3.0)
	Other	67 (4.4)

### The youth clinic survey (Paper 3)

The youth clinic survey was a cross-sectional study conducted in 20 out of the 24 youth clinics in the four northern counties of Sweden and targeting young people aged 16–25 years old. The study was conducted between September 2016 and February 2017 and data were collected by healthcare providers from 1089 participants after their visits to the youth clinics. The majority of the participants were women and had a Swedish/Scandinavian background (Table 3). The youth friendliness of the youth clinics was measured by the Youth-Friendly Health Service Swedish questionnaire and organised into 13 domains using exploratory and confirmatory factor analyses [61]. The study used multi-level analysis to assess the differences between migrant (due to the low proportion of the participants who had a migrant background, migrants here refer to first- and second-generation migrants from outside the Scandinavian countries) and Swedish/Scandinavian youths in their perceptions of youth friendliness while controlling for individual and clinic level factors. The analysis was conducted in three steps, with the first step including only the country of origin, the second step including all individual-level factors, and the third step including both individual and clinic-level factors. The results of the analysis were analysed using Stata 15 statistical software.

**Table 3.** Sample characteristics of youth clinics survey. Reprinted from [53].

		n (%)
Gender		
	Women	976 (90.9)
	Men	91 (8.5)
	Non-binary	7 (0.7)
Background		
	Swedish/Scandinavian	971 (89.2)
	Migrants	118 (10.8)
Sexual orientation		
	Hetero	926 (85.9)
	LGBTQ+	130 (12.1)
	Don’t categorise	22 (2.0)
Age		
	16 to 17	345 (32.7)
	18 to 19	324 (30.7)
	≥20	385 (36.5)

### The qualitative study (Paper 4)

The qualitative study aimed to explore the perceptions and experiences of Arabic-speaking young migrant men in Sweden regarding sexual information/education and SRH services. We recruited participants who spoke Arabic, were born in one of the Arabic countries, and moved to Sweden in the last ten years. Due to difficulties in recruiting participants younger than 29 years old, we included participants up to 37 years old. Potential participants were identified through personal contacts and those who left their contact numbers after seeing the announcement of the study on social media. They were contacted by phone to offer them verbal information about the study and then sent a written consent with information sheet. We recruited 13 participants aged between 22 and 37 years, with a mix of educational backgrounds, country of birth (Syria, Palestine, Iraq, Yemen, and Iran), and ethnicities (Arabic and Kurdish). All interviews were conducted in Arabic and took place online due to the concurrent COVID-19 pandemic from December 2020 to March 2021, with an average duration of 43 minutes and a range of 32–57 minutes. The study involved a semi-structured interview using an interview guide we developed and piloted. The interview guide included questions about the participants’ understanding of SRH, their needs, and their perceptions and experiences of SRH services (the full interview guide is published elsewhere [59]). We used a reflexive thematic analysis approach [62] to analyse the data. The interviews were first transcribed verbatim in Arabic, then reviewed and partially translated into English. The interviews were then coded in English and preliminary themes developed through discussion with co-authors. The themes were refined and renegotiated through multiple discussions, and data extracts were selected for a final analysis before presentation.

## Ethical considerations

The research process followed ethical guidelines as reported by the Belmont report, Declaration of Helsinki, and the Economic and Social Research Council (ESRC) Framework for Research Ethics [63–65]. Informed consent procedures were followed by providing participants with written information in a language they understood. The studies obtained autonomous consent from all participants, each of whom was above the legal age required for granting consent for health research in Sweden (15 years). Before providing their consent, all participants received written information about the purpose and significance of the study, the voluntary nature of their participation, the potential risks and benefits associated with participation, the confidentiality of their information, their right to withdraw from the study, and how their data would be managed. Data were managed and stored according to the guidelines of the European Union General Data Protection Regulation and the rules applied at Umeå university.

Ethical approval was granted by the Regional Ethics Committee at Umeå University [Dnr 2015-190-31O, 2017/515-31, and 2020-02816]. The research team attempted to minimise potential psychological stress in the participants by providing details about where to get information and help related to SRHR. No incentives were provided to the participants.

Culturally sensitive research is also important in conducting research with migrants, particularly in the context of SRHR [66,67]. Cultural sensitivity is defined as research approach that incorporates the cultural values, norms, beliefs, experiences, and behaviours of a specific ethnic or cultural group into its design and interpretation [68]. I, an Arabic-speaking migrant man, was involved in the formulation of the research questions and in data collection for the national survey and the qualitative study. A multi-language culturally adapted questionnaire was developed, a multicultural team was used in the national survey, and I conducted the qualitative interviews in Arabic to build trust with the participants. The research group also aimed to avoid further stigmatising migrants in the data analysis and interpretation who are often portrayed as lacking ‘Swedish-ness,’ victims of their cultures, or perpetrators in ‘Swedish’ society [32,69].

## Results and discussion

### Young migrants’ understanding and experiences of SRHR

The participants in the qualitative study conducted with Arabic-speaking men valued SRH as a crucial aspect of their lives and associated it with the ability to have children and build a family, which they considered to be a source of stability. The study participants stated that sexual health is not only physical and mental stress due to the migration process may lead migrants to ignore their SRH. The participants also linked health and rights and emphasised the relevance of obtaining accurate sexual information and education, as well as convenient SRH services. According to the participants, it is essential that these services are easily accessible and offered on equal terms to all, irrespective of their country of origin, economic status, or legal standing, which is in line with international and national conventions and acts [21,45,70].

**Figure 2.**
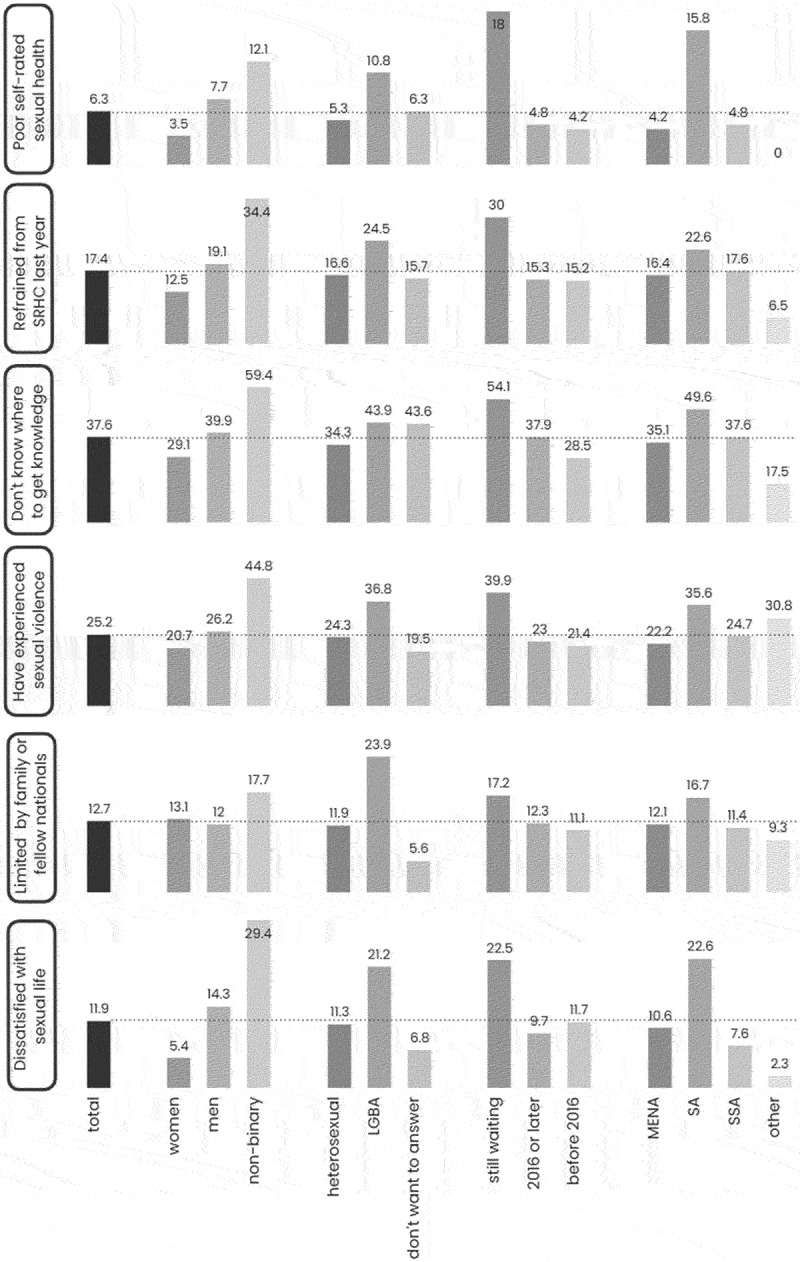
Prevalence of selected indicators of sexual rights among young migrants in Sweden. Stratified by gender, sexual orientation, residence permit and region of birth (MENA; middle East and North Africa. SA; South Asia. SSA; Sub-Saharan Africa). Reprinted from [53].

The results of the national survey showed that the majority of young migrants reported having good sexual health (76.3%), but the fulfilment of sexual rights varied between different subgroups, with men, non-binary individuals, LGBTQ+ individuals, those who were born in South Asia, and those without a residence permit reporting lower fulfilment of rights (Figure 2).

LGBTQ+ and non-binary individuals are frequently cited in the literature as being in precarious circumstances concerning SRHR [15,16,71]. Individuals who are both migrants and identify as LGBTQ+ or gender non-binary may encounter additional health disparities resulting from heterosexism and discrimination [71]. Those born in South Asia were mainly unaccompanied minors born in Afghanistan, who are known to experience poor SRH outcomes due to limited access to information, increased risk of sexual violence, and reluctance to use contraceptives because of stigma [2]. Undocumented migrants and asylum seekers are also considered highly vulnerable regarding access to health and healthcare [1].

Regarding young migrants’ experiences of their right to body integrity, the national survey showed that 25.2% of the participants reported being exposed to sexual violence, either physically, verbally, or online. Men reported being exposed to sexual violence more frequently compared to women, and the most common perpetrators were strangers, followed by partners, colleagues, and family members or friends. Unfortunately, the study did not collect information on the gender of the perpetrators. Only 30% of those who experienced sexual violence talked to someone about it, and only 3% sought help from the authorities.

Compared with the Swedish national survey, SRHR17, more young women (57%) and less young men (10%) reported sexual violence. Furthermore, more young people talked about their exposure to sexual violence and a higher percentage reported to the police [16]. The literature suggests that women are the most common victims of sexual violence among migrants and refugees [72,73]. However, some studies indicate that men and children, particularly unaccompanied boys, are also at risk of sexual violence during migration in both their home and host countries [28,74]. While women in Sweden are most affected by sexual violence, the situation of young migrant men requires more attention.

The national survey found that many young migrants experience limitations on their right to sexual autonomy, particularly in terms of choosing their intimate partners. The proportion of young migrants who reported being not limited in this respect (47.3%) was much lower than that reported in the FOKUS15 study (93%), which surveyed Swedish youth aged 16–25 years [17]. The reasons for these limitations varied, with some respondents attributing them to personal choice or adherence to religious beliefs, while others cited social norms, family, and peers from their country of origin.

Regarding the right to a safe and satisfying sexual life without stigma and discrimination, young migrants fulfiled their rights to a lesser extent compared to young people in Sweden and young migrant men fulfiled their rights to a lesser extent compared to young migrant women. A significant proportion of young migrants expressed dissatisfaction with their sexual lives (11.9%), particularly among men (14.3%) compared to women (5.4%). More men (13.6%) than women (2.6%) also stated that their most recent sexual activity took place in an unsafe location. Discrimination was another issue highlighted in the national survey, with a considerable percentage of young migrants in Sweden (36.6%) reporting that they experienced discrimination based on their ethnicity or religion during the previous year.

### Young migrants’ perceptions and experiences of SRH services

According to the national survey, nearly 30% of young migrants needed SRH services in the past year, but only 13.9% actually visited SRH services and only 10.2% had their needs met. Women were found to fulfil their SRH needs to a greater extent (52.5% of those who needed the services) compared to men (22.9%). The study found that sociodemographic factors such as gender, non-binary identity, LGBTQ+ identity, South Asian origin, lack of residence permit, and low economic status were related to challenges in accessing SRH services. The youth clinic survey also showed disparities in access to SRH services based on country of origin, with migrant youths perceiving the friendliness of the clinics as lower compared to Swedish/Scandinavian youths. The results suggest lower utilisation of SRH services among young migrants compared to the general Swedish population.

**Figure 3.**
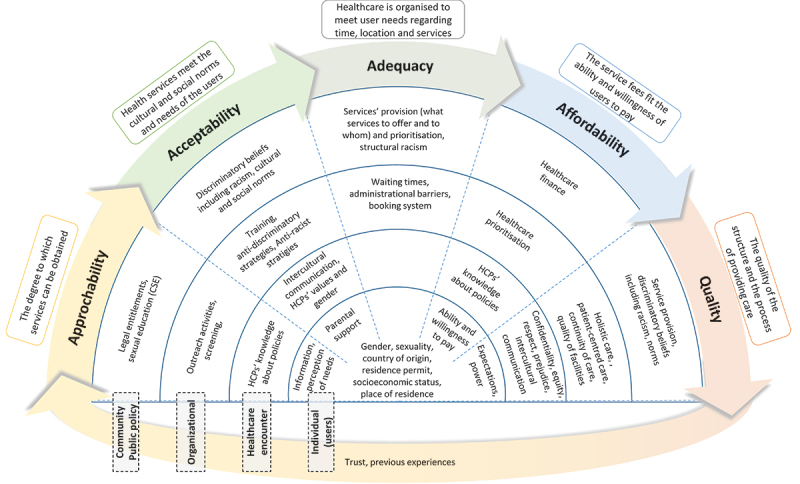
Factors related to young migrants’ access to SRH services using the proposed ecological framework of access to healthcare. Reprinted from [53].

The thesis applies the proposed framework for understanding access to SRH services among young migrants in Sweden. The framework includes individual-level factors, as well as interactions with healthcare providers, healthcare organisation, and community and public health policies. These factors can play a key role in enabling or hindering access to SRH services among migrants. The thesis highlights the opportunities and challenges in the different steps of the access process and at various levels of the ecological model (Figure 3).

#### The approachability of SRH services

The national survey showed that 17.4% of the participants refrained from visiting SRH services in the last year, around half being due to a deficiency of information about where to seek assistance, which was more prevalent among men. A large proportion (60.2%) of young migrants reported the need for more information about SRHR and sexuality, with 45.9% of them indicating that they did not know how to get new information.

The qualitative study with Arabic-speaking men also showed the need for information on where to access SRH services and the challenges of obtaining reliable sexual information online in their native language. Formal sexual education was not provided to newly arrived migrants unless they were under 20 years old and enrolled in a Swedish high school. Participants highlighted the lack of comprehensive sexual education in Sweden and in their countries of origin.

Young migrants face significant challenges in approaching SRH services in Sweden, including a lack of knowledge about where to seek help and a lack of comprehensive sexual education. This can lead to lower utilisation of SRH services among young migrants.

#### The acceptability of SRH services

According to the qualitative study, young migrants perceived Sweden as a ‘new open environment,’ which resulted in a changing attitude towards SRH issues and an increasing acceptability to seek health-care services. In their countries of origin, the participants described ‘closed’ societies that prevented young people, particularly men and unmarried women, from accessing SRH services. However, these changes in acceptability were not equally applicable to all migrants, as older migrants were less likely to use services due to their difficulty in changing their behaviours.

The youth clinic survey showed differences in perceptions of parents’ support to use youth clinics between migrant youths and Swedish/Scandinavian youths, with a lower score among migrants. The main obstacle to visiting youth clinics for both groups was the fear of being seen by parents and other adults, such as teachers and employers. This points to a broader issue of acceptability and community support, as youth require the support of parents and the community to access SRH services more easily [48].

The participants’ acceptance of education was also influenced by cultural insensitivity. Sweden’s history of referring to itself as a ‘moral superpower’ may also contribute to low acceptability of services among migrants, as researchers claim that the idea of Swedish ‘exceptionalism’ can result in an implicit bias against cultures perceived as ‘inferior’ and make it challenging to discuss issues related to racism [75,76].

In the qualitative study, the participants perceived that the education is delivered in a culturally insensitive way, with teachers and instructors not accepting ‘migrant cultures’ rather ‘indoctrinating’ them on how to be and what to do. Teaching becomes an act of using the ‘superior’ culture to indoctrinate migrants, making them feel othered and making it difficult for them to accept the new information.

The acceptability of SRH services among young migrants is, therefore, affected by several factors, including the ‘open’ environment, fear of a lack of privacy and confidentiality breaches, support from parents and the community, cultural insensitivity, and cultural racism.

#### The adequacy of SRH services

The youth clinic survey found that Swedish/Scandinavian and migrant youths considered it easy to contact youth clinics. They also considered booking an appointment within a suitable waiting time and receiving SRH help as good. However, the national survey revealed that 20% of those who refrained from visiting SRH services cited long waiting times as the reason. In the qualitative interviews with Arabic-speaking men, long waiting times were also identified as a barrier to fulfiling SRH needs. The participants also perceived that their healthcare concerns were not being taken seriously by the healthcare system. They also expressed dissatisfaction with the lack of physical examination and investigations, inadequate follow-up, and the healthcare professionals treating symptoms without attempting to diagnose the underlying causes. The participants felt that the Swedish healthcare system did not prioritise their SRH needs.

According to a recent literature review, men’s SRH services are generally given low priority in Nordic countries. This is due to a lack of knowledge and training on men’s SRH as well as insufficient support from healthcare organisation [77,78]. Migrant men’s SRH needs are further hindered by cultural insensitivity and racism, where their specific cultural needs are not taken into consideration.

#### The quality of SRH services

The national survey showed that SRH services were reported to be satisfactory by most of the young migrants who utilised them (89%), but some participants reported negative experiences. Around half the participants (45.2%) reported at least one adverse treatment related to: disrespect, discrimination, lack of privacy, inadequate help, or prejudiced attitudes of staff. This was higher among men, non-binary individuals, LGBTQ+ individuals, those without a residence permit, and those who were born in sub-Saharan Africa or South Asia.

The language barrier was also a factor in affecting the quality of the healthcare received by migrants. Many participants in the national survey (52.8%) needed an interpreter when communicating with healthcare providers, and some (7.8%) refrained from visiting SRH services due to the lack of ability to communicate in Swedish. The use of interpreters was also problematic, as participants in the qualitative study doubted their reliability and competence and felt embarrassed using interpreters from the same culture due to associating stigma with sexual health problems.

The youth clinic survey also showed that migrant youths reported lower scores compared to Swedish/Scandinavian youths in the domains of equity, respect, and quality of consultation.

Participants in the qualitative study explained their experiences of adverse treatment in different ways, one being racism. A participant reported feeling excluded in antenatal care while accompanying his wife and believed it was due to stereotypes of Eastern men as being violent and controlling. On the other hand, some participants did not blame discrimination or racism, but instead they felt not entitled to criticise the system and explained the adverse treatment as if it were a matter of individual responsibility or luck.

The exclusion of men in reproductive care could be a common issue within the healthcare system in Sweden, and studies have emphasised the need to include them as equal partners in reproductive healthcare services [77]. The acceptance of less access to healthcare due to personal responsibility, entitlement, and luck could be a manifestation of internalised racism [79].

## Methodological considerations

The main strength of this thesis is that the national survey was able to gather data through multiple modes of collection, which allowed for a large sample size and diverse representation of sociodemographic groups of young migrants but removed the possibility of conducting non-response analysis. The study was able to include participants from 56 different countries, who could read one of the six languages used in the questionnaire which were culturally and linguistically adapted. The instrument used in the youth clinic survey was tested and validated in Sweden. Additionally, evaluating the outcomes within the context of the ecological framework of access and the sexual rights domains provided valuable insights. The qualitative study was built upon the findings of the quantitative studies to understand the experiences of young migrant men in SRHR and SRH services.

However, I would like to acknowledge the difficulties faced by combining different methodologies [80]. The choice of combining qualitative and quantitative methodologies in the thesis was to enrich the results and answer the research questions from a pragmatic standpoint in the study of such complex health and social phenomena. The trustworthiness of the research was evaluated using four criteria: truth value (the ability to measure what was intended to be measured); applicability (how applicable the results are to people in similar or other contexts); consistency (repeating the study in the same context with the same participants leads to same results); and ‘neutrality’ (the researchers should distance their interests and biases from the research process) [81]. This is summarised in Table 4 for both the quantitative and qualitative parts of the thesis.

**Table 4.** Summary of trustworthiness of the qualitative and quantitative parts of the thesis. Adapted from [53].

Truth value	Quantitative part	Truth value in quantitative methodology is referred to as “internal validity” and is measured by excluding the risk of selection bias. In the national survey, the data were collected through face-to-face, online, and postal modes, which could lead to heterogeneity and bias by not reaching groups of young migrants. The combination of different data collection modes is, however, suggested to reach this “hard to reach” group. Sampling strategies for the youth clinic survey included exit interviews, which had a low proportion of migrant youths, which might be a result of selection bias. Lack of privacy in youth clinics and lack of time for completing the questionnaire are potential sources of bias.
	Qualitative part	Truth value in qualitative methodology is referred to as “credibility,” which refers to capturing the participants’ multiple realities. Being an Arabic-speaking man with migration experience, and my familiarity with the context and culture but with continuous self-reflections among the research group, helped me in understanding the realities of the researched group. The assumed similarities might have influenced the participants’ responses to meet some cultural beliefs and expectations. To mitigate this, I stated from the start of each interview that I have a professional secrecy, there are no right and wrong answers, and although I might understand what they mean, it is important that they explain their ideas in their own words to facilitate the understanding of the rest of the team. The study utilised various methods to recruit participants, such as snowball sampling and social media announcements, which helped to gather a diverse range of experiences and enriched the data. The study did not gather data on the experiences of men who have sex with men, gay men, or transmen.
Applicability	Quantitative part	Applicability in quantitative research is referred to as “generalisability.” The national survey focused on newly arrived young migrants from low- and middle-income countries, and the sample was representative of this group in terms of gender and region of origin. Therefore, the findings cannot be generalised to all young migrants in Sweden. The youth clinic survey may not reflect the experiences of those who do not use youth clinics. However, the survey did make it possible to reach a more diverse group of young migrants.
	Qualitative part	Applicability in qualitative research is referred to as “transferability.” The study provided a thorough explanation of the research context, which is important for understanding the experiences of the participants. Additionally, the study analysed the participants’ experiences using existing theories and literature, which adds to the transferability of the findings. The aim was to target specific groups of young migrants instead of generalising the results in all young migrants.
Consistency	Quantitative part	Consistency is referred to as “reliability” in quantitative research. The instrument of the national study was not validated, but strategies were used to improve its reliability. First, it was designed based on previous questionnaires, tested through a pilot study, and cultural adaptations were made. Second, a trained team familiar with multiple languages and cultures was used to collect face-to-face data for the national survey. The instrument of the youth clinic survey was validated in Sweden to ensure that the questionnaire accurately measures what it is intended to measure and is reliable over time.
	Qualitative part	Consistency in qualitative research is referred to as “dependability” which means the ability to the changes of the study context and the continuous interactions with the study participants. The study used an emergent design approach, which means that the research questions were adapted and refined based on the preliminary analysis of the data. The follow-up questions were also modified accordingly, and theoretical concepts were selected after developing potential themes from the data.
“Neutrality”	Quantitative and qualitative parts Qualitative part	Neutrality in quantitative research is referred to as “objectivity.” Neutrality in qualitative research is neither possible nor desirable since researchers should reduce the distance between them and the participants. Instead, the trustworthiness criterion to aspire for is “confirmability” which refers to enabling the readers to judge that the interpretations are grounded in the data. I think that the researcher cannot be detached from the research process, therefore, the role of the research team in the research process was discussed. The researcher’s identity and background can affect the interaction with the participants and how they understand and analyse the data. Therefore, I tried to “bracket” myself, be open to clarifications, and continuously reflected about my positionality when interacting with the participants and during the research process. The research team contributed with various perspectives about the research material.

## Concluding discussion

This thesis contributes knowledge and theory to the field of migration and sexualities. The thesis explored how young migrants understood sexual health, experienced sexual rights, and perceived and experienced access to sexuality education, information, and SRH services. The thesis also offers a useful framework to study the process of access based on the four levels of the ecological model which helps identify where the responsibility for improving access to healthcare lies.

The study showed that young migrants in Sweden considered SRH to be ‘essential’ and ‘a right.’ However, young migrants’ sexual rights were less fulfiled compared to young people in Sweden. The study also found that the sexual rights of different groups of young migrants were fulfiled to different degrees, with more adverse situations reported among men, non-binary individuals, LGBTQ+ individuals, those born in South Asia, those without a residency permit, and those who had lower economic conditions.

The study also investigated the barriers and facilitators to accessing SRH services and found that these services were largely non-*approachable*. The national survey showed that approximately half of the participants who needed SRH services did not use them because they lacked information about them. The youth clinic survey suggested that migrant youths had low utilisation of youth clinics. The perceived ‘open’ environment of Sweden was found to increase the *acceptability* of seeking SRH services, although several factors hindered this, including insufficient cultural sensitivity, less parental support, and a fear of exposure to adults. In addition, concerns about the *adequacy* of services were highlighted, for example, the long waiting times and the perception that SRH services were not taking their issues seriously, which could indicate cultural racism. Young migrants described the quality of healthcare as good; perceived youth clinics as youth friendly; and were satisfied with the services they received, as 89% of respondents in the national survey reported satisfaction with services. However, negative experiences were also reported by almost half of those who used the services. These negative experiences were reported in the domains of respect, equity, privacy, non-prejudice, and quality of consultation. Additionally, lower scores in the equity, respect, and quality of consultation domains in youth clinics were reported.

In Sweden, most SRH services are legally accessible to young migrants, the services are of good quality and are available, and there is an ‘open’ environment in Sweden that makes SRH services accessible for young migrants. However, there are several factors that limit the accessibility of these services.

Young migrants face a significant barrier to seeking healthcare in the form of limited information on sexuality, SRH, and how to access healthcare [82,83]. Although the state is responsible for providing comprehensive sexual education to its citizens, this opportunity is often missed in their countries of origin creating a gap in their knowledge about SRH. Sexual education for young migrants in Sweden is almost only provided to those who are young enough to go to ordinary high schools (usually younger than 20 years). Additionally, education is sometimes delivered from a starting point of ‘exceptional’ and ‘modern’ Swedish culture which contributes to othering and stigmatising young migrants [75,76,84].

Furthermore, language barriers contribute to limited access to SRH services, as around 50% of the participants in the national survey needed an interpreter when visiting healthcare or other authorities. The literature points out various issues related to the availability of high-quality interpreting services in Sweden [85]. Furthermore, the lack of language proficiency among some migrants is used to marginalise them and view them as being ‘in deficit’ [86,87]. This discrimination necessitates assimilation, where migrants are expected to acquire language skills, knowledge about the Swedish culture, and be ‘open’ to change in order to be included [86,87].

Culture plays a critical role in SRH services’ acceptability. The participants in the qualitative study perceived the ‘open’ culture of Sweden as a facilitating factor for accepting SRH services. At the same time migrants’ cultures are often used as an explanation for poor SRH among young migrants. This ‘open’ versus ‘closed’ culture should be problematised. The ‘open’ environment of Sweden is often touted as being progressive when it comes to sexuality and SRH. However, there are still taboos and gendered prejudices that exist in Swedish culture [88] and there are variations between the cultures in Sweden [89,90] as well as between migrant cultures. The division between ‘us’ and ‘them’ is fuzzy and cultural racism in healthcare can lower trust and discourage young migrants from seeking and adhering to treatment [91]. Cultural racism here refers to the belief that one culture is inherently ‘superior’ to others. This belief is then used to justify disparate treatment for people of ‘inferior’ culture and is demonstrated with stereotypes and prejudices which the participants reported in both the qualitative and quantitative parts of the study [92]. When this belief is held by the people of the ‘inferior’ culture, the term internalised racism is used which was demonstrated in the qualitative study particularly in the context of participants blaming themselves for the negative experiences they encountered in SRH services [79].

The proposed ecological framework of access acknowledges that other factors beyond the healthcare system may also contribute to health outcomes. The framework, therefore, deliberately excludes health as an outcome. There are multiple factors that affect the health outcomes of migrants, including their contexts, vulnerabilities, the power structure of the hosting society, and how this power structure is embedded in the practices and policies of its public institutions [93–95]. These factors play a role in creating the conditions of young migrants and their access to resources and a risk for violations of their rights.

## Policy and research recommendations

The national strategy for SRHR in Sweden prioritises young people and migrants as key groups in need of improved SRHR [21]. To improve the SRHR of young migrants, there are several recommendations aimed at increasing the approachability, acceptability, adequacy, and quality of SRH services. To increase approachability, young migrants should have access to information about SRH services and comprehensive sexual education in their native language and in a culturally sensitive manner. Improving acceptability can be achieved by improving the intercultural communication of HCPs and the cultural sensitivity of the services without contributing to ‘othering’ migrants, handling the confidentiality concerns, and building trust in the healthcare system. The adequacy of SRH services should consider the SRH needs of all including young migrants and men. To improve quality, the healthcare system must be free from racism and discrimination, and an action plan should be developed to ensure this. Finally, the focus should not be on the migrants themselves but on addressing the structural factors affecting their contexts and vulnerabilities, such as unequal social conditions, racism, and gender inequality. Policies and strategies to improve these conditions and challenge power structures and gender norms can improve SRHR among young men and women.

There is a need for future research related to the SRHR of young migrants including developing and evaluating interventions to improve access to information, increase cultural competency of HCPs, and improve quality of interpreters and cultural mediators. Research is also needed to assess experiences of young migrant men exposed to sexual violence and investigate the effects of cultural racism on migrants.

## Paper context

We conducted a mixed method study to explore young migrants’ sexual and reproductive health which is understudied in Sweden. We found higher sexual rights’ violations and less access to services among young migrants. The access was facilitated by the ‘open environment’ and the availability of good quality services; however, serious barriers exist such as limited access to information concerning the health system, comprehensive sexual education, lack of cultural sensitivity, and cultural racism.

## References

[1] World Health Organization. Report on the health of refugees and migrants in the WHO European region: no public health without refugee and migrant health. Copenhagen: WHO Regional Office for Europe; 2018. Available from: http://www.euro.who.int/__data/assets/pdf_file/0004/392773/ermh-eng.pdf?ua=1

[2] Mason-Jones AJ, Nicholson P. Structural violence and marginalisation. The sexual and reproductive health experiences of separated young people on the move. A rapid review with relevance to the European humanitarian crisis. Public Health. 2018;158:156–17. doi: 10.1016/j.puhe.2018.03.009 29653866

[3] European Centre for Disease Prevention and Control. Migrant health: HIV testing and counselling in migrant populations and ethnic minorities in EU/EEA/EFTA member states. 2011.

[4] Knight M, Kurinczuk JJ, Spark P, Brocklehurst P. Inequalities in maternal health: national cohort study of ethnic variation in severe maternal morbidities. BMJ. 2009;338:b542. doi: 10.1136/bmj.b542 19261591 PMC2654771

[5] Botfield JR, Zwi AB, Newman CE. Young migrants and sexual and reproductive healthcare. In: Handbook of migration and health. Edward Elgar Publishing; 2016.

[6] Marston C, King E. Factors that shape young people’s sexual behaviour: a systematic review. Lancet. 2006;368:1581–1586. doi: 10.1016/S0140-6736(06)69662-1 17084758

[7] Cha E. Inequalities and multiple discrimination in access to and quality of healthcare. 2013.

[8] Mengesha ZB, Dune T, Perz J. Culturally and linguistically diverse women’s views and experiences of accessing sexual and reproductive health care in Australia: a systematic review. Sex Health. 2016;13:299–310. doi: 10.1071/SH15235 27209062

[9] Keygnaert I, Ivanova O, Guieu A, Van Parys A-S, Leye E, Roelens K. What is the evidence on the reduction of inequalities in accessibility and quality of maternal health care delivery for migrants? A review of the existing evidence in the WHO European region. Regional Office For Europe: World Health Organization; 2016. 27786434

[10] Baroudi M, Goicolea I, Hurtig A-K, San-Sebastian M. Social factors associated with trust in the health system in northern Sweden: a cross-sectional study. BMC Public Health. 2022;22. doi: 10.1186/s12889-022-13332-4 PMC9065232 35509072

[11] Rechel B, Mladovsky P, Ingleby D, Mackenbach JP, McKee M. Migration and health in an increasingly diverse Europe. Lancet. 2013;381:1235–1245. doi: 10.1016/s0140-6736(12)62086-8 PubMed PMID: WOS:000317350100034. 23541058

[12] Buttigieg S Summary report on the MIPEX health strand and country reports. Geneva: International Organization for Migration; 2016. p. 9290687312.

[13] Paradies Y, Ben J, Denson N, et al. Racism as a determinant of health: a systematic review and meta-analysis. PLoS One. 2015;10:e0138511. doi: 10.1371/journal.pone.0138511 26398658 PMC4580597

[14] Hamed S, Thapar-Björkert S, Bradby H, Ahlberg BM. Racism in European health care: structural violence and beyond. Qual Health Res. 2020;30:1662–1673. doi: 10.1177/1049732320931430 32546076 PMC7410275

[15] Folkhälsomyndigheten. Sexualitet och hälsa bland unga i Sverige – UngKAB15 – en studie om kunskap, attityder och beteende bland unga 16–29 år. Stockholm; 2017. Available from: https://www.folkhalsomyndigheten.se/contentassets/11272529714342b390d40fe3200f48cf/sexualitet-halsa-bland-unga-sverige-01186-2017-1-webb.pdf

[16] Folkhälsomyndigheten. Sexuell och reproduktiv hälsa och rättigheter (SRHR) i Sverige 2017 – Resultat från befolkningsundersökningen SRHR 2017. Stockholm; 2019. Available from: https://www.folkhalsomyndigheten.se/publikationer-och-material/publikationsarkiv/s/sexuell-och-reproduktiv-halsa-och-rattigheter-i-sverige-2017/

[17] Myndigheten för ungdoms- och civilsamhällesfrågor (MUCF). FOKUS15. Ungas sexuella och reproduktiva rättigheter: En tematisk kartläggning. Stockholm: Myndigheten för ungdoms- och civilsamhällesfrågor; 2015. Available from: https://www.mucf.se/sites/default/files/2016/01/fokus15_0.pdf

[18] Yin Z, Brown AE, Rice BD, Marrone G, Sönnerborg A, Suligoi B, et al. Post-migration acquisition of HIV: estimates from four European countries, 2007 to 2016. Eurosurveillance. 2021;26:2000161. doi: 10.2807/1560-7917.ES.2021.26.33.2000161 34414881 PMC8380976

[19] Socialstyrelsen. Ojämna villkor för hälsa och vård – Jämlikhetsperspektiv på hälso- och sjukvården. [Unequal conditions for health and healthcare - equity perspective in health health care]. Stockholm: Socialstyrelsen; 2011.

[20] Danielsson M, Berglund T, Forsberg M, Larsson M, Rogala C, Tydén T. Sexual and reproductive health: health in Sweden: the national public health report 2012. Chapter 9. Scand J Soc Med. 2012;40:176–196. doi: 10.1177/1403494812459600 23238407

[21] Folkhälsomyndigheten. Nationell strategi för sexuell och reproduktiv hälsa och rättigheter (SRHR); En god, jämlik och jämställd sexuell och reproduktiv hälsa i hela befolkningen. Folkhälsomyndigheten; 2020. Available from: https://www.folkhalsomyndigheten.se/contentassets/0d489b0821164e949c03e6e2a3a7e6cc/nationell-strategi-sexuell-reproduktiv-halsa-rattigheter.pdf

[22] The Global Migration Group (GMG). Migration and youth: challenges and opportunities 2014. Available from: https://unesdoc.unesco.org/ark:/48223/pf0000227720

[23] Ungdomsmottagningen online (UMO). Vad behöver unga nyanlända? En studie av ensamkommandes och andra unga nyanländas behov när det gäller frågor kring sexuell och reproduktiv hälsa och rättigheter (SRHR) och psykisk hälsa. 2016. Available from: https://www.umo.se/globalassets/umo/block/standardtexter/umo-rapport-vad-behover-unga-nyanlanda.pdf

[24] Wagenius CM, San Sebastian M, Gustafsson PE, Goicolea I. Access for all? Assessing vertical and horizontal inequities in healthcare utilization among young people in northern Sweden. Scand J Public Health. 2019;47:1–8. doi: 10.1177/1403494818774965 29779450

[25] Hopkinson RA, Keatley E, Glaeser E, Erickson-Schroth L, Fattal O, Nicholson Sullivan M. Persecution experiences and mental health of LGBT asylum seekers. J Homosex. 2017;64:1650–1666. doi: 10.1080/00918369.2016.1253392 27831853

[26] Keygnaert I, Vettenburg N, Temmerman M. Hidden violence is silent rape: sexual and gender-based violence in refugees, asylum seekers and undocumented migrants in Belgium and the Netherlands. Cult Health Sex. 2012;14:505–520. doi: 10.1080/13691058.2012.671961 22468763 PMC3379780

[27] Folkhälsomyndigheten. Migration, sexuell hälsa och prevention : två kunskapsöversikter med fokus på risktagande och riskutsatthet i samband med migration. Stockholm: Smittskyddsinstitutet (SMI); 2012. Available from: https://www.folkhalsomyndigheten.se/pagefiles/12840/migration-prevention-sexuell-halsa.pdf

[28] Freccero J, Biswas D, Whiting A, Alrabe K, Seelinger KT. Sexual exploitation of unaccompanied migrant and refugee boys in Greece: approaches to prevention. PLOS Med. 2017;14:e1002438. doi: 10.1371/journal.pmed.1002438 29166401 PMC5699806

[29] Chynoweth SK, Freccero J, Touquet H. Sexual violence against men and boys in conflict and forced displacement: implications for the health sector. Reprod Health Matters. 2017;25:90–94. doi: 10.1080/09688080.2017.1401895 29227205

[30] Statistics Sweden (SCB). Invandring till sverige [Migration to Sweden] 2021. Available from: https://www.scb.se/hitta-statistik/sverige-i-siffror/manniskorna-i-sverige/invandring-till-sverige/

[31] Wernesjö U. Across the threshold: negotiations of deservingness among unaccompanied young refugees in Sweden. J Ethn Migr Stud. 2020;46:389–404. doi: 10.1080/1369183X.2019.1584701

[32] Herz M. ‘Becoming’a possible threat: masculinity, culture and questioning among unaccompanied young men in Sweden. Identities. 2019;26:431–449. doi: 10.1080/1070289X.2018.1441692

[33] Socialstyrelsen & Folkhälsomyndigheten. Underlag till nationell strategi för sexuell och reproduktiv hälsa och rättigheter. Stockholm: Socialstyrelsen; 2014. Available from: https://www.socialstyrelsen.se/globalassets/sharepoint-dokument/artikelkatalog/kunskapsstod/2014-10-26.pdf

[34] Justitiedepartementet. Förvaltningslag § 13 (2017:900), [Administration Act]. L6 J, editor; 2017.

[35] Socialdepartementet. Smittskyddslag (2004:168), 7 kap. Ersättning, [Communicable Diseases Act]. Socialdepartementet, editor; 2004.

[36] FSUM [Föreningen för Sveriges ungdomsmottagningar]. Handbok för Sveriges ungdomsmotagningar. [Handbook of Sweden’s youth clinics]. 2018. Available from: http://www.fsum.nu/wp-content/uploads/2018/05/handbok_original_utskrift.pdf

[37] Författningssamling S. Hälso-och sjukvårdslag. SFS. 1982;763. https://www.riksdagen.se/sv/dokument-och-lagar/dokument/svensk-forfattningssamling/halso-och-sjukvardslag-1982763_sfs-1982-763/

[38] Sverige Regeringen. God och jämlik hälsa – en utvecklad folkhälsopolitik [Good and equal health - a developed public health policy]. In: Socialdepartementet, editor. Regeringens proposition 2017/18:249. Stockholm; 2018. https://www.regeringen.se/rattsliga-dokument/proposition/2018/04/prop.-201718249/

[39] Akhavan S. Midwives’ views on factors that contribute to health care inequalities among immigrants in Sweden: a qualitative study. Int J Equity Health. 2012;11:47. doi: 10.1186/1475-9276-11-47 22900923 PMC3462105

[40] Nielsen SS, Hempler NF, Waldorff FB, Kreiner S, Krasnik A. Is there equity in use of healthcare services among immigrants, their descendents, and ethnic Danes? Scand J Public Health. 2012;40:260–270. doi: 10.1177/1403494812443602 22637365

[41] Hammarström S, Alehagen S, Kilander H. Violence and sexual risk taking reported by young people at Swedish youth clinics. Ujms. 2022;127:127. doi: 10.48101/ujms.v127.7823 PMC8788656 35140876

[42] Christianson M, Boman J, Essen B. “Men don’t think that far” - interviewing men in Sweden about chlamydia and HIV testing during pregnancy from a discursive masculinities construction perspective. Sex Reprod Healthc. 2017;12:107–115. doi: 10.1016/j.srhc.2017.03.007 Epub 2017/05/10. PubMed PMID: 28477922. 28477922

[43] Sollesnes R. Exploring issues that motivate contact with adolescent health. Vård i Norden. 2010;30:4–7. doi: 10.1177/010740831003000202 PubMed PMID: 10083099.

[44] United Nations Department of Economic and Social Affairs (UNDESA). The 17 goals | sustainable development 2015. Available from: https://sdgs.un.org/goals

[45] Starrs AM, Ezeh AC, Barker G, Basu A, Bertrand JT, Blum R, et al. Accelerate progress—sexual and reproductive health and rights for all: report of the Guttmacher–Lancet Commission. Lancet. 2018;391:2642–2692. doi: 10.1016/S0140-6736(18)30293-9 29753597

[46] Evans DB, Hsu J, Boerma T. Universal health coverage and universal access. Bullet World Health Organ. 2013;91:546–A. doi: 10.2471/BLT.13.125450 PMC3738317 23940398

[47] Kowalski S. Universal health coverage may not be enough to ensure universal access to sexual and reproductive health beyond 2014. Glob Public Health. 2014;9:661–668. doi: 10.1080/17441692.2014.920892 24938423

[48] World Health Organization. Adolescent friendly health services: an agenda for change: Geneva: World Health Organization; 2003. Available from: http://www.who.int/iris/handle/10665/67923

[49] World Health Organization. Making health services adolescent friendly: developing national quality standards for adolescent friendly health services. Geneva: WHO; 2012. p. p.

[50] Obrist B, Iteba N, Lengeler C, Makemba A, Mshana C, Nathan R, et al. Access to health care in contexts of livelihood insecurity: a framework for analysis and action. PLoS Med. 2007;4:e308. doi: 10.1371/journal.pmed.0040308 17958467 PMC2039761

[51] Levesque J-F, Harris MF, Russell G. Patient-centred access to health care: conceptualising access at the interface of health systems and populations. Int J Equity Health. 2013;12:18. doi: 10.1186/1475-9276-12-18 23496984 PMC3610159

[52] Bronfenbrenner U. The ecology of human development: experiments by nature and design. Cambridge (Massachusetts) and London (England): Harvard university press; 1979.

[53] Baroudi M Leaving the door ajar : young migrants’ sexual and reproductive health in Sweden [Doctoral thesis, comprehensive summary]. Umeå: Umeå universitet; 2022.

[54] Goddard M, Smith P. Equity of access to health care services: theory and evidence from the UK. Soc Sci Med. 2001;53:1149–1162. doi: 10.1016/S0277-9536(00)00415-9 11556606

[55] Ivankova NV, Creswell JW, Stick SL. Using mixed-methods sequential explanatory design: from theory to practice. Field Methods. 2006;18:3–20. doi: 10.1177/1525822X05282260

[56] Baroudi M, Hurtig A-K, Goicolea I, San Sebastian M, Jonzon R, Nkulu-Kalengayi FK. Young migrants’ sexual rights in Sweden: a cross-sectional study. BMC Public Health. 2021;21:1–13. doi: 10.1186/s12889-021-11672-1 34482819 PMC8420038

[57] Baroudi M, Nkulu Kalengayi F, Goicolea I, Jonzon R, San Sebastian M, Hurtig A-K. Access of migrant youths in Sweden to sexual and reproductive healthcare: a cross-sectional survey. Int J Health Policy Manag. 2022;11:287–298. doi: 10.34172/ijhpm.2020.123 32729283 PMC9278465

[58] Baroudi M, San Sebastian M, Hurtig A-K, Goicolea I. The perception of youth health centres’ friendliness: does it differ between immigrant and Swedish-Scandinavian youths? Eur J Public Health. 2020;30:780–785. doi: 10.1093/eurpub/ckaa077 32417877 PMC7445032

[59] Baroudi M, Goicolea I, Hurtig A-K. The good, the bad, and the why: how do Arabic-speaking migrant men perceive and experience information and services related to sexual and reproductive health in Sweden? J Migr Health. 2023;7:100153. doi: 10.1016/j.jmh.2023.100153 36798098 PMC9926105

[60] Erens B, Phelps A, Clifton S, Mercer CH, Tanton C, Hussey D, et al. Methodology of the third British national survey of sexual attitudes and lifestyles (Natsal-3). Sex Transm Infect. 2014;90:84–89. doi: 10.1136/sextrans-2013-051359 24277881 PMC3933071

[61] Baroudi M, Waenerlund A-K, San Sebastian M, Goicolea I. Assessing the dimensionality of YFHS-Swe: a questionnaire to assess youth-friendliness in differentiated health services. Global Health Action. 2017;10:1380399. doi: 10.1080/16549716.2017.1380399 29043946 PMC5678427

[62] Braun V, Clarke V. Using thematic analysis in psychology. Qual Res Psychol. 2006;3:77–101. doi: 10.1191/1478088706qp063oa

[63] The National Commission for the Protection of Human Subjects of Biomedical and Behavioral Research. The Belmont report: ethical principles and guidelines for the protection of human subjects of research. Washington; 1978. 25951677

[64] World Medical Association. World Medical Association Declaration of Helsinki. Ethical principles for medical research involving human subjects. Bullet World Health Organ. 2001;79:373. doi: 10.4414/fms.2001.04031 PMC2566407 11357217

[65] Economic and Social Research Council. ESRC framework for research ethics: updated January 2015. 2015.

[66] Awad GH, Patall EA, Rackley KR, Reilly ED. Recommendations for culturally sensitive research methods. J Educ Psychol Consult. 2016;26:283–303. doi: 10.1080/10474412.2015.1046600

[67] Düvell F, Triandafyllidou A, Vollmer B. Ethical issues in irregular migration research in Europe. Popul Space Place. 2010;16:227–239. doi: 10.1002/psp.590

[68] Burnette CE, Sanders S, Butcher HK, Rand JT. A toolkit for ethical and culturally sensitive research: an application with indigenous communities. Ethics Soc Welf. 2014;8:364–382. doi: 10.1080/17496535.2014.885987

[69] Branteryd F, Gallo C, Brown E, Svensson K. Crime victims, immigrants and social welfare: creating the racialized other in Sweden. Br J Criminol. 2021;62:948–964. doi: 10.1093/bjc/azab089

[70] Sverige Regeringen. En förnyad folkhälsopolitik. In: Socialdepartementet, editor. Regeringens proposition 2007. Stockholm: Regeringen; 2008. p. 110.

[71] Zeeman L, Sherriff N, Browne K, McGlynn N, Mirandola M, Gios L, et al. A review of lesbian, gay, bisexual, trans and intersex (LGBTI) health and healthcare inequalities. Eur J Public Health. 2019;29:974–980. doi: 10.1093/eurpub/cky226 30380045 PMC6761838

[72] JdO A, FMd S, Proença R, Bastos ML, Trajman A, Faerstein E. Prevalence of sexual violence among refugees: a systematic review. J Revista de saude publica. 2019;53:78. doi: 10.11606/s1518-8787.2019053001081 PMC6752644 31553381

[73] De Schrijver L, Vander Beken T, Krahé B, Keygnaert I, health p. Prevalence of sexual violence in migrants, applicants for international protection, and refugees in Europe: a critical interpretive synthesis of the evidence. Int j Environ Res Public Health. 2018;15:1979. doi: 10.3390/ijerph15091979 30208610 PMC6165364

[74] Landberg Å, Jonsson L, Svedin CG Den långa resan - möten med barn som misstänks vara utsatta för människohandel och sexuell exploatering [Elektronisk resurs]. Länsstyrelsen i Stockholms län; 2015. Available from: https://catalog.lansstyrelsen.se/store/39/resource/2015__17

[75] Dahl A-S. Sweden: once a moral superpower, always a moral superpower? Int J. 2006;61:895–908. doi: 10.1177/002070200606100408

[76] Ahlberg BM, Hamed S, Thapar-Björkert S, Bradby H. Invisibility of racism in the global neoliberal era: implications for researching racism in healthcare. Front Sociol. 2019;4:61. doi: 10.3389/fsoc.2019.00061 33869384 PMC8022523

[77] Baroudi M, Stoor JP, Blåhed H, Edin K, Hurtig A-K. Men and sexual and reproductive healthcare in the Nordic countries: a scoping review. BMJ Open. 2021;11:e052600. doi: 10.1136/bmjopen-2021-052600 PMC8487177 34593504

[78] Grandahl M, Bodin M, Stern J. In everybody’s interest but no one’s assigned responsibility: midwives’ thoughts and experiences of preventive work for men’s sexual and reproductive health and rights within primary care. BMC Public Health. 2019;19:1423. doi: 10.1186/s12889-019-7792-z Epub 2019/11/02. Epub 2019/11/02 31666036 PMC6822360

[79] Banks KH, Stephens J. Reframing internalized racial oppression and charting a way forward. Soc Issues Policy Rev. 2018;12:91–111. doi: 10.1111/sipr.12041

[80] Bowling A. Research methods in health: investigating health and health services. Berkshire: McGraw-Hill Education (UK); 2014.

[81] Dahlgren L, Lindhgren M, Emmelin M, Hällgren Graneheim U, Sahlén KG, Winkvist A. Qualitative methodology for international public health. 3rd ed. Umeå: Department of Epidemiology and Global Health, Umeå University; 2019.

[82] Graetz V, Rechel B, Groot W, Norredam M, Pavlova M. Utilization of health care services by migrants in Europe—a systematic literature review. Br Med Bull. 2017;121:5–18. doi: 10.1093/bmb/ldw057 28108435

[83] Åkerman E. Challenges and opportunities for sexual and reproductive healthcare services for immigrant women in Sweden. Uppsala: Acta Universitatis Upsaliensis; 2019.

[84] Bredström A. ‘Love in another country’-‘race’, gender and sexuality in sexual education material targeting migrants in Sweden. Sexualities. 2005;8:517–535. doi: 10.1177/1363460705056624

[85] Krupic F, Hellström M, Biscevic M, Sadic S, Fatahi N. Difficulties in using interpreters in clinical encounters as experienced by immigrants living in Sweden. J Clin Nurs. 2016;25:1721–1728. doi: 10.1111/jocn.13226 26879885

[86] Fejes A, Dahlstedt M. Popular education, migration and a discourse of inclusion. Stud Educ Adults. 2017;49:214–227. doi: 10.1080/02660830.2018.1463656

[87] Sharif H. “Här i Sverige måste man gå i skolan för att få respekt”: Nyanlända ungdomar i den svenska gymnasieskolans introduktionsutbildning. Uppsala (Sweden): Acta Universitatis Upsaliensis; 2017.

[88] Linander I, Goicolea I, Wiklund M, Gotfredsen A, Strömbäck M. Power and subjectivity: making sense of sexual consent among adults living in Sweden. NORA-Nord J Fem Gend Res. 2021;29:110–123. doi: 10.1080/08038740.2021.1903553

[89] Jansson A. The hegemony of the urban/rural divide: cultural transformations and mediatized moral geographies in Sweden. Space Cult. 2013;16:88–103. doi: 10.1177/1206331212452816

[90] Daun Å. Swedish mentality. USA: University Park; 2021.

[91] Ben J, Cormack D, Harris R, Paradies Y, Zeeb H. Racism and health service utilisation: a systematic review and meta-analysis. PLoS One. 2017;12:e0189900. doi: 10.1371/journal.pone.0189900 29253855 PMC5734775

[92] Taguieff P-A. The force of prejudice: on racism and its doubles. Minneapolis (USA): University of Minnesota Press; 2001.

[93] Matlin SA, Depoux A, Schütte S, Flahault A, Saso L. Migrants’ and refugees’ health: towards an agenda of solutions. Public Health Rev. 2018;39:1–55. doi: 10.1186/s40985-018-0104-929450102

[94] Marmot M. Just societies, health equity, and dignified lives: the PAHO equity commission. Lancet. 2018;392:2247–2250. doi: 10.1016/S0140-6736(18)32349-3 30262335

[95] Solar O, Irwin A. A conceptual framework for action on the social determinants of health. Geneva (Switzerland): WHO Document Production Services; 2010.

